# Cross-linking mass spectrometry for mapping protein complex topologies *in situ*

**DOI:** 10.1042/EBC20220168

**Published:** 2023-03-29

**Authors:** Kitaik Lee, Francis J. O'Reilly

**Affiliations:** Center for Structural Biology, Center for Cancer Research, National Cancer Institute (NCI), Frederick, MD 21702-1201, U.S.A.

**Keywords:** Crosslinking, in situ, protein-protein interactions, proteomics

## Abstract

Cross-linking mass spectrometry has become an established technology to provide structural information on the topology and dynamics of protein complexes. Readily accessible workflows can provide detailed data on simplified systems, such as purified complexes. However, using this technology to study the structure of protein complexes *in situ*, such as in organelles, cells, and even tissues, is still a technological frontier. The complexity of these systems remains a considerable challenge, but there have been dramatic improvements in sample handling, data acquisition, and data processing. Here, we summarise these developments and describe the paths towards comprehensive and comparative structural interactomes by cross-linking mass spectrometry.

## Introduction

The study of protein–protein interactions is essential to understand how cells function and for developing effective treatments for disease. For this, the bridging of different levels of detail, from system-wide protein interaction screens to structures solved at near-atomic resolution is necessary. Cross-linking mass spectrometry (cross-linking MS) is one technique that has the potential to bridge these scales.

Cross-linking MS is a structural proteomics technique that can produce topological information on proteins at single residue resolution. Due to its compatibility with heterogeneous and lowly abundant samples, it can be applied in a wide variety of contexts. It is this versatility for protein structure analysis that has made it an important part of the integrative structural biology toolkit [1,2]. Cross-linking MS data has helped reveal the architecture of many protein complexes essential to life, mostly purified *in vitro* [3–10]. However, many protein interactions are very difficult to reconstitute and so must be studied *in situ*. While the range of possible applications of cross-linking MS ranges from single proteins to whole tissues, the more complex samples still present significant technical challenges.

The basic principle of cross-linking MS is that nearby protein residues can be cross-linked together and after digestion, those cross-linked protein residues can be identified, thus revealing proximity in the original sample. The maximum length of the cross-linking reagent acts as a distance restraint that informs on the 3D structure. An experiment comprises cross-linking reaction, protein digestion, enrichment of cross-linked peptides, mass spectrometric acquisition, database search, and error rate control. The described pipeline comes in many flavours and like all mass spectrometry-based technologies has been subject to significant improvements in recent years [11].

In this review, we will discuss how cross-linking MS has overcome difficulties found when studying protein complexes *in situ*, and assess how close we are to comprehensively map the structural interactomes of cells and tissues using this technique.

## Fundamental challenges for cross-linking MS in complex systems

**Figure 1:**
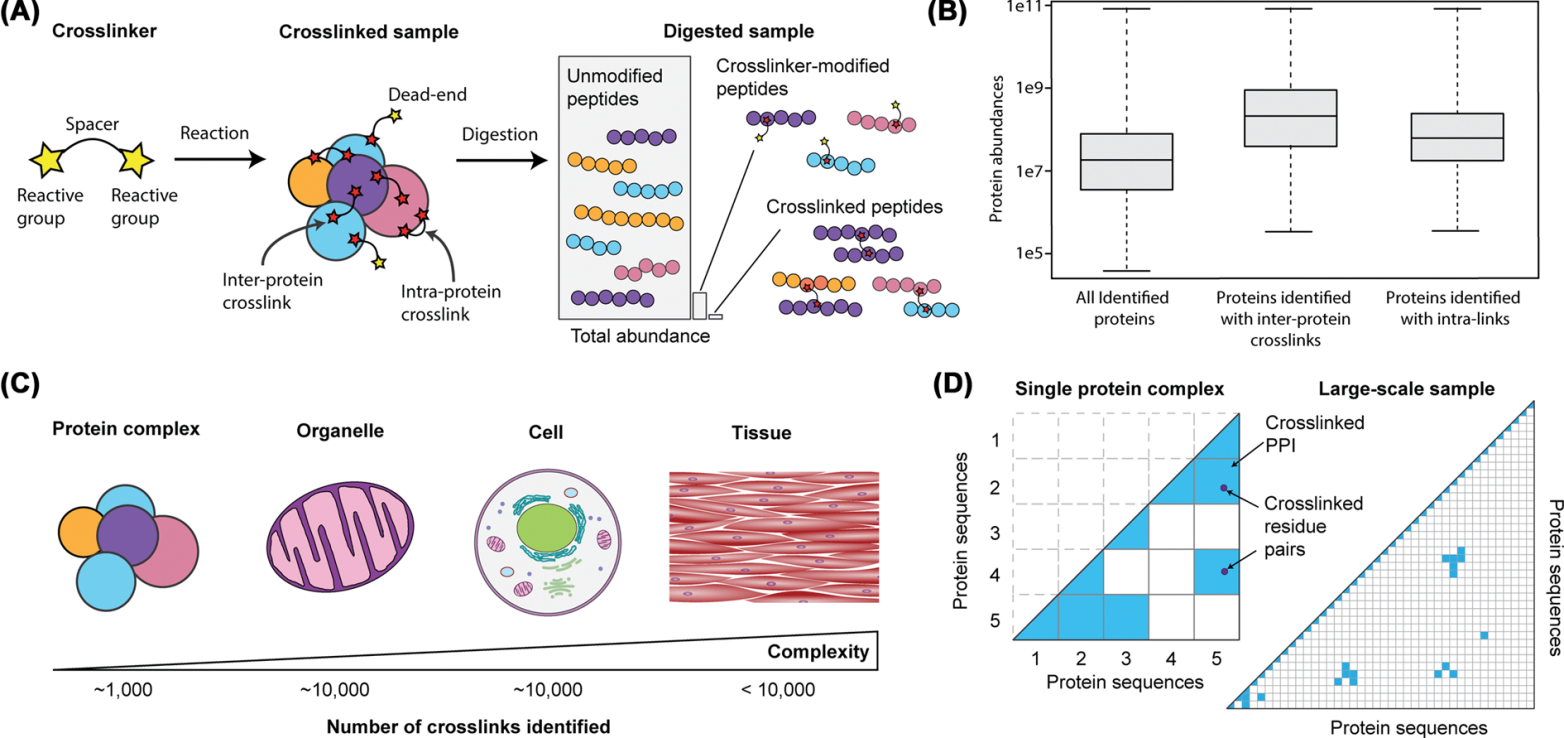
Challenges for identifying cross-linked peptides from complex samples (**A**) Cross-linkers are made up of two reactive groups and a spacer that dictates the maximum distance between the two cross-linked residues. After the cross-linked sample is digested the cross-linked peptides make up a small percentage of the total mass in the sample. (**B**) In large-scale samples containing 1000s of proteins cross-links have only been detected from the most abundant proteins. Intra-protein cross-links tend to be easier to identify than inter-protein cross-links. (**C**) All biological complexities have been studied with cross-linking MS but the coverage of the proteomes in all complex samples is poor. (**D**) The number of peptide combinations to be searched in purified complexes is tiny compared with all the possible combinations in samples containing thousands of proteins. This means that for these more complicated samples the fragmentation spectra must be more detailed to confidently identify the cross-linked peptides.

Several ambitious studies have attempted to produce interaction maps in intact cells using cross-linking MS with significant investments of mass spectrometry resources. They have found that identified cross-linked peptides are highly biased towards the most abundant proteins and protein interactions [12–15] (Figure 1). This is evident when looking at gross numbers of cross-links identified. In purified protein complexes, samples may yield over 1000 cross-linked residue pairs identified [16], while the number of cross-links identified in studies of entire cells has been limited to ∼10,000 [17] (Figure 1). This is a consequence of two related challenges of identifying *in situ* cross-links.

First, cross-linked peptides are sub-stoichiometric compared to the ‘background’ of un-cross-linked peptides. Indeed, it has been shown that cross-linked peptides make up a tiny fraction of the digested mixture, as low as <0.1% by ion intensity [17,18] (Figure 1). This is true for purified complexes but it becomes even more of a problem when considering the range of protein abundances in native samples, where cross-linkers preferentially react with abundant proteins [19]. These comparatively rare cross-linked peptides are therefore often not selected for fragmentation by the mass spectrometer. There are fundamental issues with mass spectrometry that contribute to this problem, including LC (liquid chromatography) column capacity, ion trapping capacity, and signal-to-noise limit of the detector. Therefore, cross-linked peptides need to be significantly enriched prior to injection on the mass spectrometer.

Second, the fragmentation spectrum of a cross-linked peptide is a convolution of peaks from two peptides. Specialized matching software is used to identify these two peptides from this fragmentation data. For purified complexes the number of potential cross-linked peptide pairs is relatively small so weaker spectra can still provide enough information for a confident match. As the number of possible peptides in the database increases, their pairwise combinations increase quadratically (n-squared problem) (Figure 1D). Spectra from complex samples must contain enough information to confidently match two peptides from an enormous number of combinations. In conclusion, successful matching of spectra in a large-scale search is hindered by cross-linked species with lower abundances, combined with a simultaneous increase in search space that requires improved spectra quality. As we will see, each step in the cross-linking MS workflow can be optimized to try to overcome these problems.

## Strategies for enriching cross-links from complex samples

**Figure 2:**
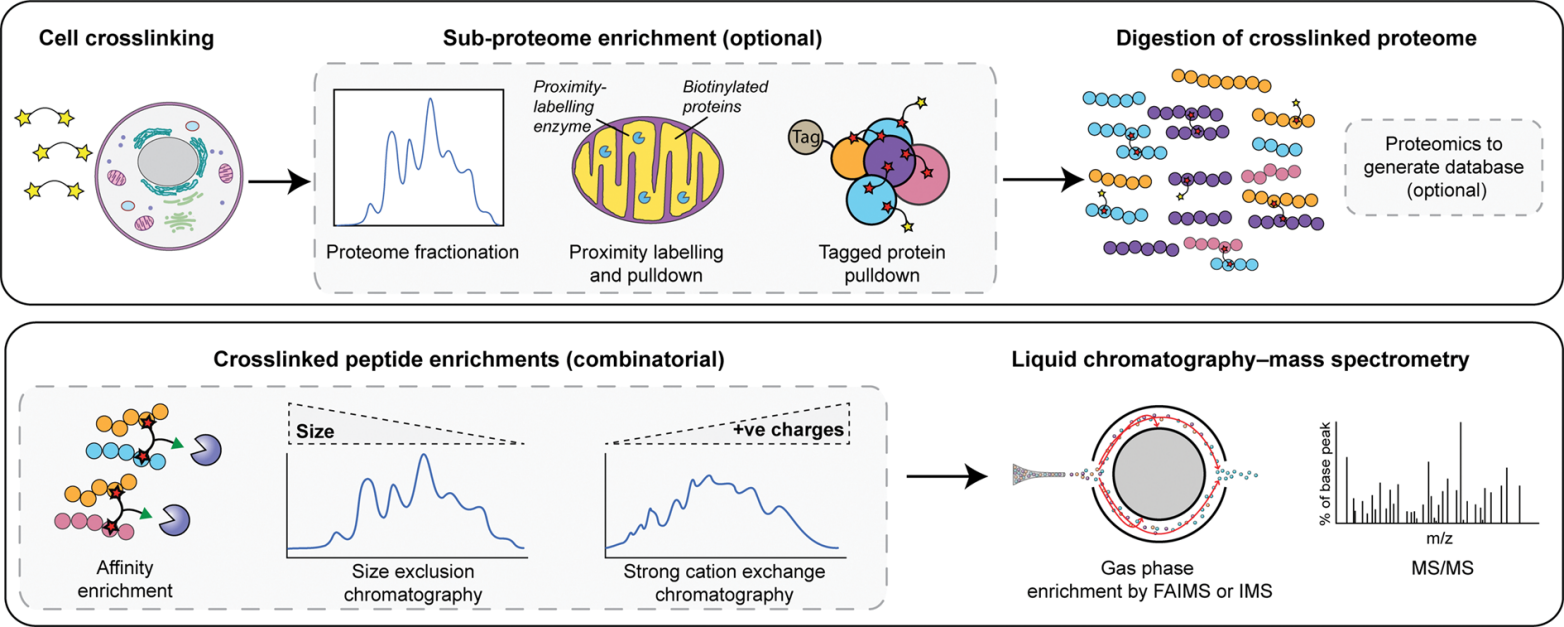
In cell cross-linking MS pipeline for complex samples (**A**) Whole cells can be cross-linked and the whole proteome can be digested for cross-linking MS analysis. To boost the signal from proteins of interest the cross-linked proteome can be simplified prior to digestion. These simplified proteomes can be analysed by standard proteomics to identify the proteins and simplify the database searched for cross-linking MS. (**B**) Cross-linked peptides can be enriched from digested peptide mixtures by affinity enrichment and/or chromatography. Cross-linked peptides can be further enriched in the gas phase by FAIMS or by an ion mobility cell prior to MS/MS analysis

Several studies have attempted to generate entire cellular or organellar interactomes by cross-linking the entire proteome for digestion and analysis. While this approach allows for the most novel interactions to be discovered, the starting material is as complicated as is possible. Depending on the particular target, the easiest way to deal with the overwhelming cellular complexity is to enrich the interactome of interest from the cross-linked cells before digestion, reducing the number of proteins to be considered (Figure 2). Studies have targeted different aspects by either; fractionating the cross-linked proteome [20], biotinylating of all proteins in certain cellular compartments [21], or genetically tagging specific proteins of interest with a suitable tag for pull-down [22–24]. The covalent cross-links formed retain the interaction information even in very harsh purification conditions.

**Figure 3:**
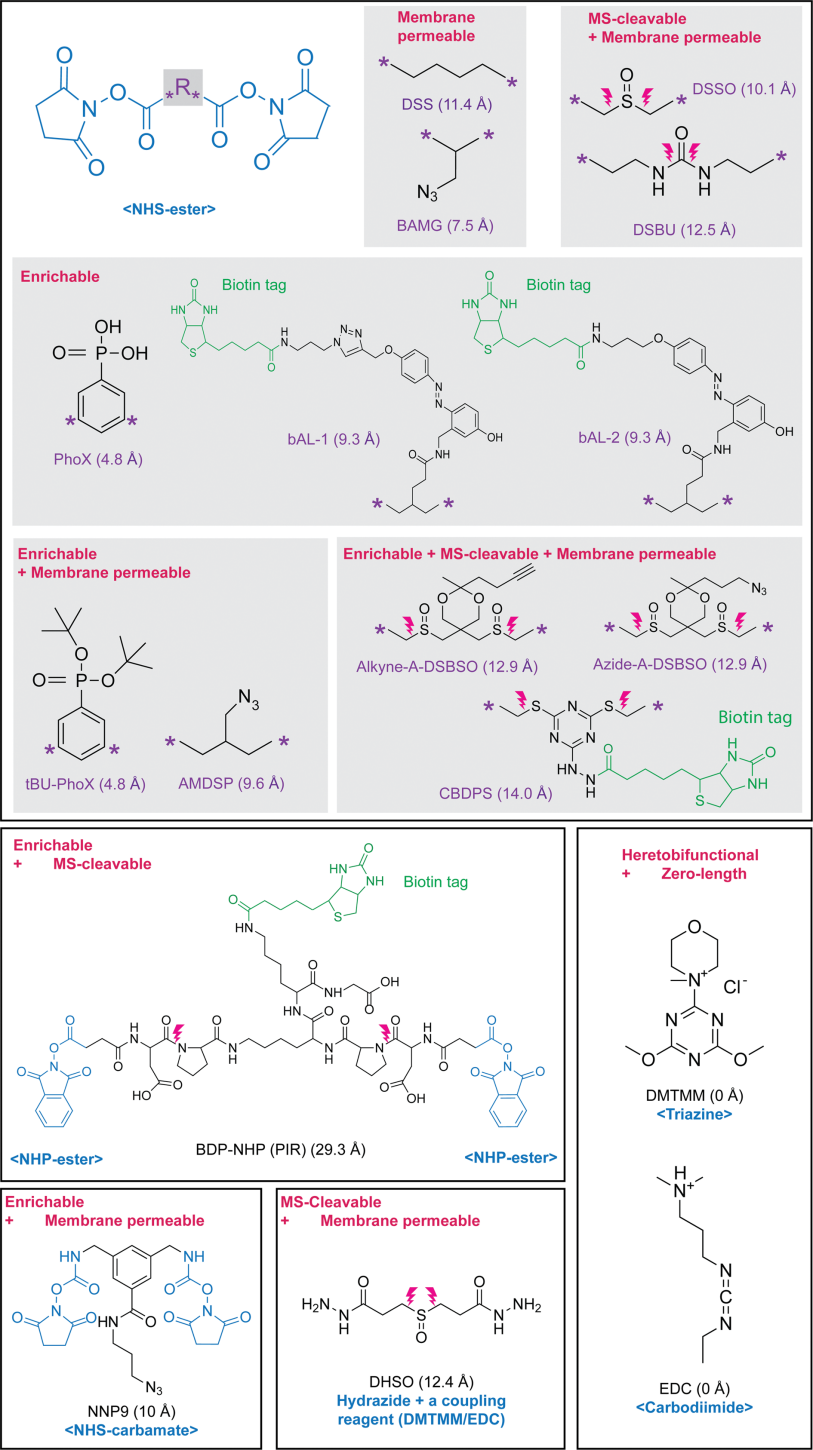
Cross-linkers successfully applied to high complexity samples, and their associated reactive group, features, and backbone spacer distances [32–36]

The choice of cross-linker will affect many aspects of the downstream processing and is an important consideration for *in situ* studies (Figure 3). For simple purified protein complexes − where the goal is to produce as many cross-links to use as distance restraints for structural modelling – cross-linking MS protocols allow for several cross-linker chemistries and features. However, for analysing a protein complex in cells, the cross-linker needs to be able to cross a cell membrane and have very specific reactivities to limit the potential combinations of cross-linked residues, while simultaneously retaining the native structure. Depending on the complexity of the sample, the cross-linker may require additional functionality in its spacer that allows specific enrichment of cross-linked peptides or aids identification in the mass spectrometer.

The most common cross-linkers for large-scale studies are homo-bifunctional and use N-hydroxysuccinimide (NHS) esters as the reactive groups. NHS-esters are highly reactive with lysines and the N-termini of proteins, with some activity towards serine, threonine, and tyrosine [25,26]. They are also specific, reactive at physiologic conditions, and easily quenched. Other chemistries that target other chemical groups such as thiols (cysteines) or carboxylic acids (aspartate, glutamate, or protein C-terminus) are available [11,27] (Figure 3), but they are seldom used for *in situ* studies. Ideally, a cross-linker would react with any residue and would therefore provide the most varied structural information. However, such an unspecified cross-linker would cause a huge search space problem for identification even for simple samples. Instead, for simplified systems, a compromise is to use a hetero-bifunctional cross-linker such as SDA (succinimidyl 4,4′-azipentanoate), which has an NHS-ester on one side and an ‘unspecific’ diazirine on the other side, which is activated with UV light. The UV-activated carbene is active for a very short time and therefore produces many fewer structural artefacts than homo-bifunctional NHS-esters [28]. However, due to the dilution in signal (due to the larger number of possible cross-links that can be formed) and a further increase in search space, these cross-linked peptides are often more difficult to identify. Only recently have some proof-of-concept studies shown that it is possible to use these cross-linkers in complex samples [29]. Translationally incorporated diazirine-containing photo-activatable amino acids are also available [30,31]. These allow probing of the interior of proteins and patches inaccessible to soluble cross-linking reagents; however, the search space and signal-dilution problems remain.

As mentioned, one of the fundamental challenges in cross-linking mass spectrometry is to enrich the cross-linked peptides enough so that they can be selected for fragmentation in the mass spectrometer. Offline enrichment is common, with size exclusion chromatography enriching on the larger Stoke’s radius of cross-linked peptides [37], and strong cation exchange enriching on the higher number of positive charges on cross-linked peptides [38–41]. Both of these techniques provide only modest enrichment, though they still greatly increase the number of identified cross-linked sites. These have been used together for 2D enrichment [14,15,20] or in combination with high-pH fractionation [42] or anion exchange fractionation [43].

A much more specific approach is to produce trifunctional cross-linkers that contain an additional reactive group that aids the enrichment of cross-linked peptides after protein digestion (Figure 3). Cross-linkers have been synthesised that contain biotin [44–48,33,34], azide moieties [49–52,32,36], or alkyne moieties [17,52–54] in their spacer region. Another approach is to use spacer chemistry that can be enriched chromatographically; cross-linkers that contain an extra positive charge that can aid enrichment by strong cation exchange [55], or cross-linkers that contain a phosphonic acid that can be enriched with IMAC chromatography [18,56]. Further offline fractionation of peptides is still required after any specific enrichment of cross-linked peptides to remove a large amount of cross-linker-modified (or dead-end) peptides that will be co-enriched. Therefore, a lot of starting material is often still required (Figure 2).

Finally, cross-linked peptides can be enriched in the gas phase after injection into the mass spectrometer. There have been studies showing that the physicochemical properties of cross-linked peptides allow for them to be separated from other species using the FAIMS device [57,58] or ion mobility cells [59] (Figure 2). This removes highly abundant species that prevent cross-links from being selected for fragmentation and also reduces co-isolation events where two overlapping precursor ions are fragmented producing a messy MS2 spectrum.

## Strategies for identifying cross-linked peptides from complex samples

As in standard proteomics, the database search software generates theoretical spectra from protein sequences and compares them with the experimental spectra in order to identify peptides. Therefore, the LC-MS acquisition strategy, the database search strategy, and the subsequent error estimations are all intimately linked and need careful consideration when handling complex samples.

**Figure 4:**
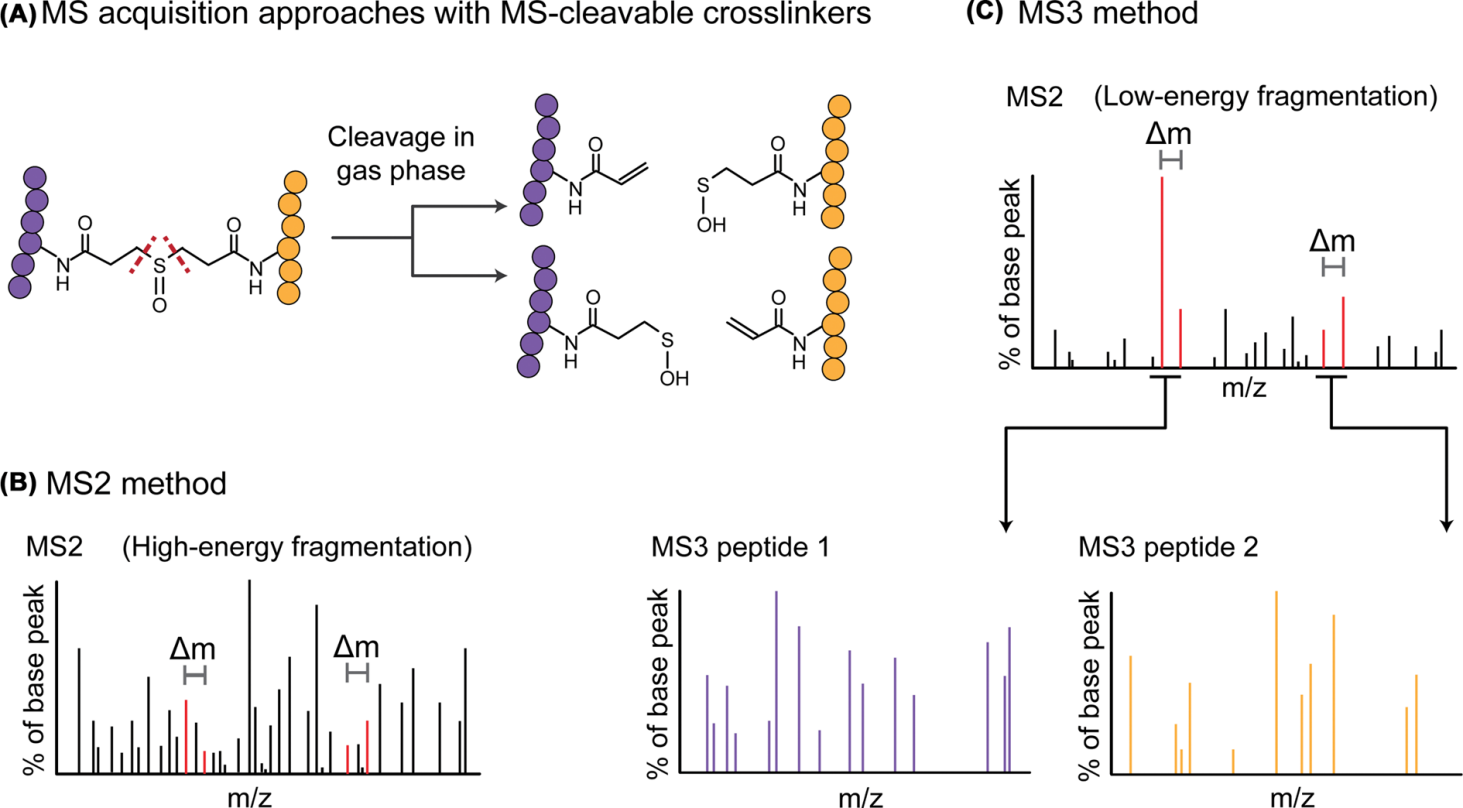
MS-cleavable cross-linkers aid identification (**A**) The DSSO spacer can be cleaved asymmetrically to produce distinctive fragment doublets in MS2. (**B**) the ‘MS2 method’ is to match cross-linked peptides from the MS2 spectra and later check the matches by confirming that the doublets exist for that combination of peptide masses. (**C**) The MS3 approach is to gently cleave the cross-linker and reveal the two peptides based on the doublet masses. These can then be further fragmented to identify the individual peptides.

A common problem with the identification of cross-linked spectra matches, especially from large databases, is the unequal fragmentation of the two linked peptides, often with one fragmenting very poorly [60]. A method to address this problem is to use MS2-cleavable cross-linkers that allow much better fragmentation of both peptides [61]. Additionally, MS2-cleavable cross-linkers that cleave asymmetrically produce characteristic ‘doublet’ peaks in the MS2 spectra that can reveal the masses of the two linked peptides. This feature can be used in two ways, each peptide can be selected and further fragmented using MS3 to provide cleaner spectra for peptide identification [62–65], and the doublets can be used to confirm the mass of each cross-linked peptide within the MS2 spectra [35,66,67] (Figure 4). Excitingly, there now exist cross-linkers that are both affinity-enrichable and MS-cleavable [17,52,68,69] (Figure 3).

There are other options that have been suggested to improve the information content of the fragmentation spectra. Each MS1 precursor can be fragmented by multiple complementary MS2 fragmentation methods to combine their information for database search [67,70]. Each MS1 and MS2 spectrum should be collected at as high resolution as feasible, which will practically reduce the search space by using tight mass tolerances when matching peaks [71]. Finally, intensities of lowly abundant but useful peaks in the MS2 spectra can be boosted by increasing ion injection times. Unfortunately, none of these options allow for ‘fast’ acquisitions, so many cross-linked peptides will simply not be selected for fragmentation. For this reason, repeated injections of the same samples are usually very valuable.

**Figure 5:**
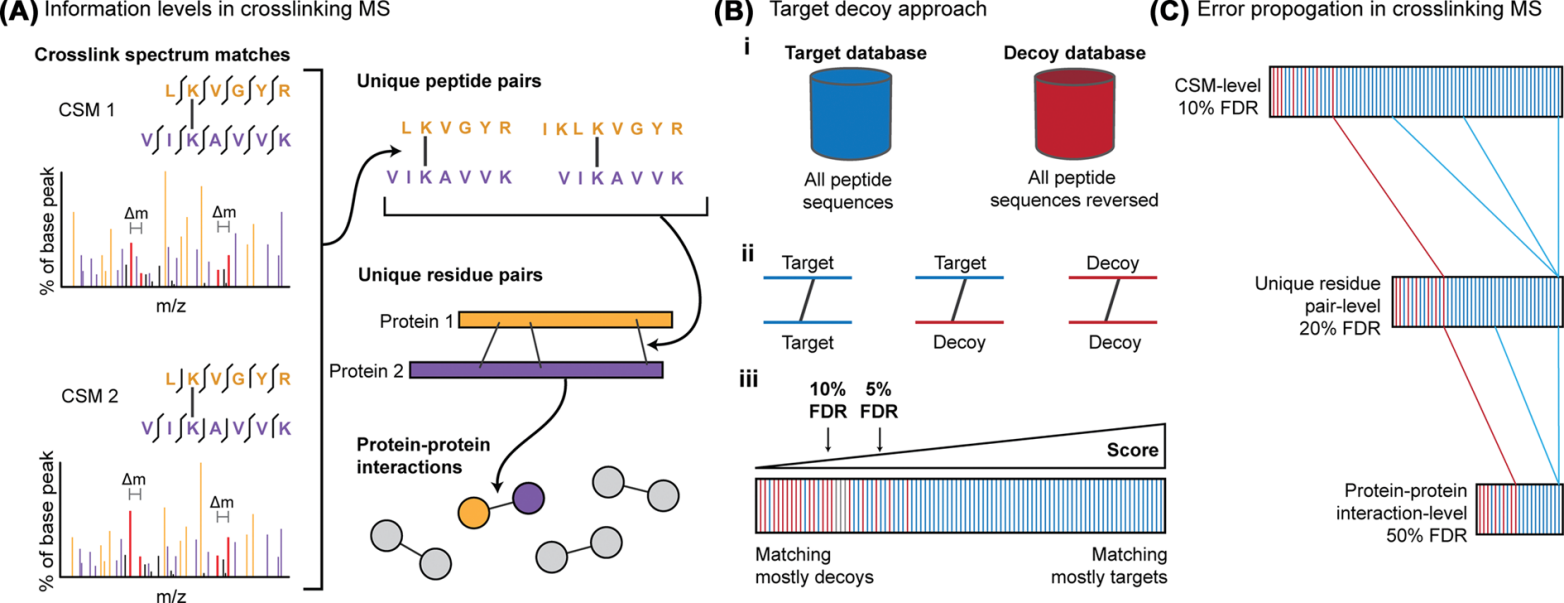
Error control for cross-linking MS (**A**) Cross-linking data contains multiple levels of information. Multiple CSMs can represent a single unique cross-linked peptide pair. Multiple cross-linked peptide pairs can represent a single unique residue pair and many unique residue pairs can represent one PPI. (**B**) **i** – The target-decoy approach is where each spectrum is searched against a ‘target’ database containing all the protein sequences, and against a ‘decoy’ database of false sequences of the same size and composition as the target database. **ii** – In cross-linking MS each CSM can be a combination of targets and decoys. **iii** – Matches to the decoy database are used as a proxy for noise and can be used to estimate the false discovery rate at any score threshold. (**C**) Error propagates through the information levels so the FDR should be estimated at the information level required for the study. False CSMs do not consolidate throughout the levels so if FDR is estimated at CSM-level, the error rate at PPI-level can be very high. The solution is to merge to the desired level (PPI-level, for examples) prior to calculating the FDR.

There are numerous different successful software solutions currently available that use different methods for identifying cross-linked peptides from fragmentation spectra, too many to review here [11]. However, accurate error estimation and thresholding of the results have proven to be full of pitfalls [43,72–74]. The typical way of estimating the false discovery rate (FDR) in cross-linking MS, as in regular proteomics, is the target decoy approach where incorrect peptide sequences are included in the search as a model for noise [75]. Cross-linking MS has a few extra quirks above linear proteomics where one or both peptides can be matched as a decoy [76] (Figure 5). It has also been shown that intra- and inter-linked peptides need to be separated for error estimation due to their different potential error spaces [43,77,78]. The error can then be propagated through the different levels of information from cross-link spectrum matches (CSM) to peptide pair to residue pair to PPI [43,76] (Figure 5). When dealing with protein interaction maps generated by cross-linking MS, minor error rates on matched CSMs can sum up to large numbers of false PPIs, making the interaction networks nearly uninterpretable if no additional FDR control is performed at the PPI level [43,72]. Different software have different mechanisms to generate and handle decoys, which do not always provide an accurate representation of the error, therefore we recommend entrapment databases as a trivial way to check [43].

Finally, bespoke visualisation software has been produced to aid the interpretation of all of the different levels of cross-linking MS data. These include matched spectra, protein interaction networks, and residue-level data that contains the structural information to be mapped on structures [79,80]. Each of these levels should be integrated when designing follow-up studies to validate the PPIs discovered.

## Discovering protein–protein interactions *in situ*

Cross-linking MS studies on proteins in their native context usually have the goal of an unbiased survey of protein interactions. Protein interaction screens are important for discovering novel biology and many such screens have been performed with techniques such as two-hybrid methods, affinity purification MS, proximity mapping, or co-fractionation MS. These other technologies have provided us with the majority of the PPIs we know today. However, they each have their limitations and so cellular interactomes remain incomplete. Cross-linking MS has unique advantages for protein interaction discovery; it can investigate any part of the cell, and labile interactions can be fixed prior to analysis.

Several studies have now demonstrated *in situ* cross-linking MS of whole bacteria [15,20,81–84], isolated organelles [12–14,85–88], plant cells [89], mammalian cells [17,56,90,91], and heart tissue [92]. However, as discussed, the bias towards the most abundant protein complexes does mean that large amounts of these interactomes are still missed. For example, in bacterial cells, a lot of identified interactions involve heat shock proteins due to their relative ubiquity. A large concentration of cross-links are typically identified on very abundant complexes such as the RNA polymerase, the ribosome, or the ATP synthase, while most other interactions are identified with only one or two residue pairs each [15,20,81–84]. These, therefore, provide very limited structural information for the majority of the PPIs identified. Despite this limitation exciting novel biology has been discovered and validated in these limited bacterial interactomes [15,20,82].

If we look at the mitochondria cross-linking MS studies of, an example of an interaction that was unlikely to be discovered by more traditional PPI-mapping technologies is apoptosis-inducing factor 1 binding to complex IV of the respiratory chain [87,93]. This type of interaction is likely reliant on its cellular context, including membranes and organellar compartmentalization and so is best studied *in situ*. Cross-linking MS not only discovered this interaction but also provided distance restraints to allow the likely interaction surface to be modelled [93].

So far almost all studies have been performed with homo-bifunctional NHS-ester cross-linkers dissolved in solvents like DMSO or dimethylformamide and added to the cells. However, it has been shown that NHS-ester cross-linkers fail to penetrate far into large cells before reacting, leaving much of the cell non-cross-linked [94]. On the other hand, it was shown to be possible to detect cross-linked peptides when performing the cross-linking reaction in minced tissue [92]. We do not know of a study that has thoroughly investigated an ‘optimal’ cross-linker concentration for cross-linking proteins *in situ*. Too little cross-linker and the cross-links will be undetectable, too much and the proteins become challenging to digest, presumably as the very interconnected proteome provides steric hindrance to proteases. It may be that there is no optimal amount of cross-linker to detect cross-links on all proteins in cells, so multiple concentrations will have to be investigated [19].

**Figure 6:**
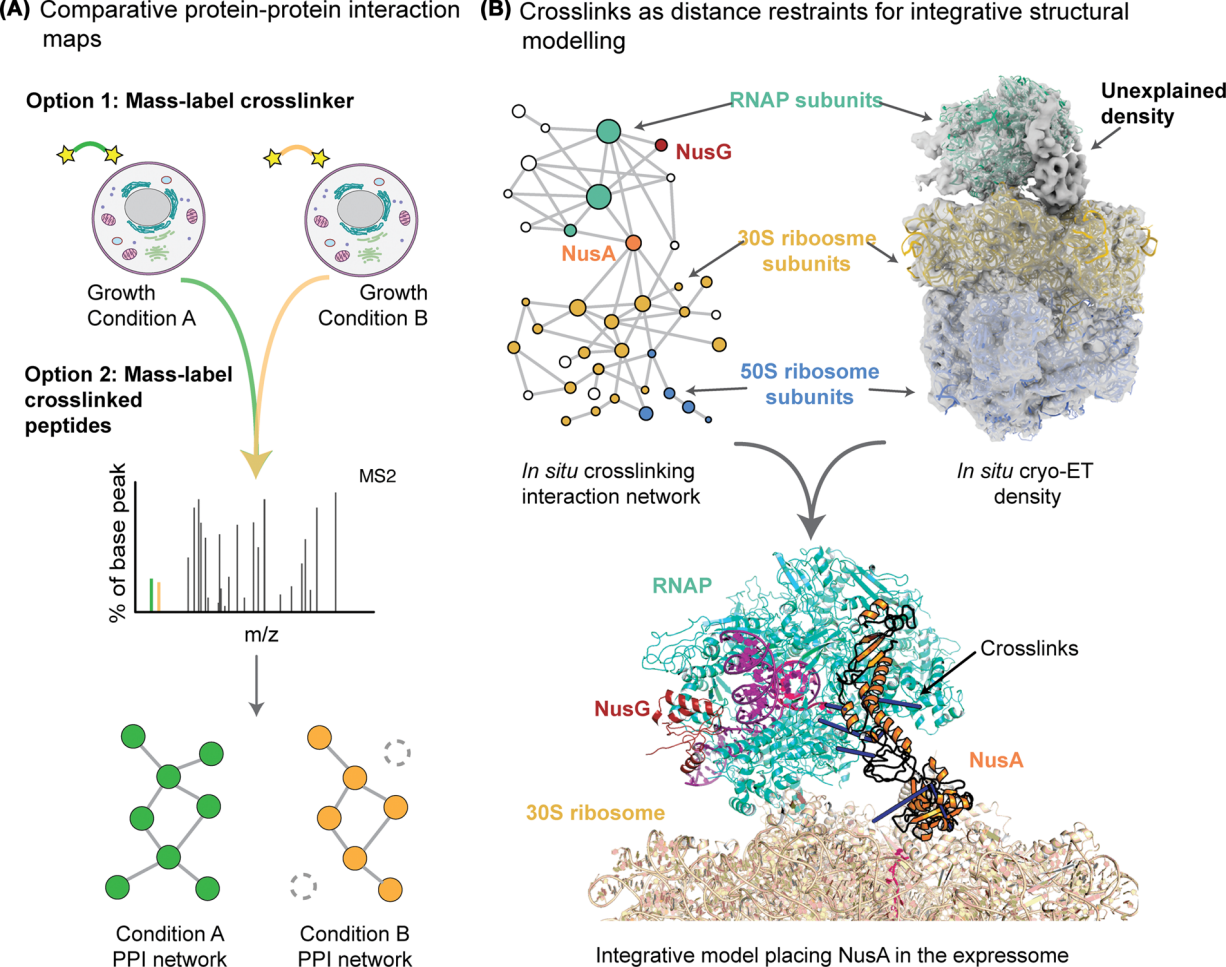
Biological discovery with *in situ* cross-linking MS data (**A**) Comparative or ‘quantitative’ cross-linking MS can compare abundances of cross-links formed in different samples. There are several ways to incorporate the mass differences that are identified in the mass spectrometer; they can either be in the cross-linker or added to peptides after digestion. (**B**) An interaction network including subunits of the RNA polymerase (RNAP) and the ribosome was identified by cross-linking intact cells. In parallel, whole-cell cryo-electron tomography of ribosomes produces a density that shows an RNAP and some unexplained other density bound to the 30S ribosome. Together these data, the electron density and the cross-links as distance restraints, could be combined into an integrative model that placed NusA into the unexplained density.

The ‘static’ interaction maps generated in the studies mentioned above can suggest potential functions of the identified PPIs based on previous knowledge. A future challenge will be to adapt this approach to yield quantitative estimates of how PPIs change in different cellular conditions. Comparative or ‘quantitative’ cross-linking MS is a very powerful tool to investigate protein conformation changes in purified protein complexes [95] but is still very challenging in complex systems. Proof-of-concept studies have attempted to map interactome changes induced by a range of drugs [90,96–98] or changes of carbon source [99]. These large-scale comparative experiments have relied on additional labelling and tagging approaches: peptides may be labelled with tandem mass tags (TMT) [100] or SILAC [90], or the cross-linker contains an isobaric label that is liberated during fragmentation [101] (Figure 6). Again, the low intensity of the cross-linked peptides imposes significant limitations on these studies as not all of the identified links and interactions can be quantified.

## Cross-links as *in situ* structural information for model building

As we have discussed, the advantage of cross-linking MS over other interaction methods is that identified links contain structural information in addition to the reported interaction. Cross-links can be used as distance restraints in protein structure modelling or validation as the spacer dictates the maximum distance between the two cross-linked residues. In studies on purified complexes, this information has helped model building into cryo-EM maps by assigning densities, threading novel folds, assigning registers and mapping interfaces [4]. How one can use these distance restraints to model protein structures for purified complexes has been reviewed in depth [1]. The coverage of distance restraints generated from *in situ* data is much more limited. Still, this structural information often implies structure–function relationships that can allow targeted validation experiments to be designed [20,102].

A great deal of structural information already exists for many cellular complexes, so even sparse distance restraints generated from *in situ* cross-linking MS can roughly localize novel interfaces [93]. If there are enough distance restraints, all of the structural information can be combined in integrative modelling software suites such as the Integrative Modelling Platform [103], HADDOCK [104], or Rosetta [105], to produce plausible structural models of the proposed interfaces. These approaches rely on integrating experimental restraints with available structural information, be it experimental or structure prediction approaches, to come up with an integrative model of assemblies. The advent of accurate predictions of protein structures by AlphaFold2 and RosettaFold increases the power of these approaches by providing an accurate library of ‘building blocks’ for integrative models.

In addition to integrative modelling approaches, the increased power of protein structure prediction in addressing multi-protein complexes mean that novel PPIs discovered by cross-linking MS can be structurally predicted, with predictions independently validated by fitting of models to the experimental distance restraints [20,106,107]. Cross-linking MS can be combined with other techniques to bring structural biology approaches directly *in situ*, in order to study the structure and function of molecular machines in their native environment. A combination that has proven especially powerful is the integration of cross-linking MS with cryo-electron tomography (cryo-ET), which can generate structures of large protein complexes in their native states and sometimes reveal unexpected molecular architectures [108]. However, the resolution of the electron maps is often too low to confidently identify the proteins belonging to the densities. Cross-linking MS data can be used to assign these densities, bringing structural biology closer to the native state of the complexes [15,109]. In one notable example, these techniques were combined to discover the architecture of the super-complex that couples transcription and translation in the bacterium *Mycoplasma pneumoniae*. This supercomplex had an unexplained density between RNAP and the ribosome that could be confidently assigned to the transcription elongation factor NusA with the integration of cross-linking MS data (Figure 6). It is becoming apparent that, in conjunction with AI-powered structure prediction tools, these approaches have the potential of revealing both the structure and dynamics of biological systems inside cells.

## Conclusions

As we have discussed, while cross-linking MS for purified complexes is an established technology for deriving residue–residue distances, it is a much more difficult proposition to use it to map protein interactions in intact cells. The main limitation is the abundance range of the proteome and the relative scarcity of the cross-links formed. We have shown that with clever experimental design, current pipelines can produce exciting biological discoveries and such studies are becoming more ambitious. Moreover, there are still innovations used in other proteomics workflows that have yet to be integrated into cross-linking MS pipelines, such as an improved understanding of cross-linked peptide fragmentation patterns or elution time predictions. Further technological innovations across the pipeline will carry us towards the goal of rapid and comparative structural interactomes, where we know there is still much to be discovered [110].

## Summary

- Unlike for purified complexes, cross-linking mass spectrometry for analysing protein interactions *in situ* is still a technological frontier.
- Several studies have overcome experimental and computational hurdles and made important discoveries about the structure of protein interactions in cells.
- Further developments will allow for comprehensive and comparative analysis of the structural interactomes of difficult-to-study complexes *in situ*.

## Abbreviations

AI: artificial intelligence
AMDSP: azide-tag-modified disuccinimidyl pimelate
bAL-1: biotinylated Azo-Leiker 1
bAL-2: biotinylated Azo-Leiker 2
BAMG: bis(succinimidyl)-3-azidomethyl glutarate
BDP-NHP: biotin aspartate proline-N-hydroxyphthalamide
CBDPS: cyanurbiotindipropionylsuccinimide
cryo-EM: cryogenic electron microscopy
cryo-ET: cryogenic electron tomography
CSM: cross-link spectrum-match
DHSO: dihydrazide sulfoxide
DMSO: dimethyl sulfoxide
DMTMM: 4-(4,6-dimethoxy-1,3,5-triazin-2-yl)-4-methylmorpholinium chloride
DSBCO: disuccinimidyl bis-sulfoxide
DSBU: disuccinimidyl dibutyric urea
DSS: disuccinimidyl suberate
DSSO: disuccinimidyl sulfoxide
EDC: 1-ethyl-3-[3-dimethylaminopropyl]carbodiimide hydrochloride
FAIMS: field asymmetric waveform ion mobility spectrometry
FDR: false discovery rate
IMAC: immobilized metal affinity chromatography
IMS: ion mobility spectrometry
LC: liquid chromatography
MS: mass spectrometry
MS/MS: tandem mass spectrometry
NHP: N-hydroxyphthalamide
NHS: N-hydroxysuccinimide
PhoX: disuccinimidyl Phenyl Phosphonic Acid,
PIR: protein interaction reporter
PPI: protein-protein interaction
RNAP: RNA polymerase
SILAC: stable isotope labeling by amino acids in cell culture
tBU-PhoX: tert-Butyl Disuccinimidyl Phenyl Phosphonate
TMT: tandem mass tags
